# Functional roles and mechanisms of circRNA-protein interactions in cancer progression and tumor immune regulation

**DOI:** 10.3389/fimmu.2026.1771949

**Published:** 2026-02-11

**Authors:** Lin Yang, Chunhong Li, Xiulin Jiang, Yixiao Yuan, Chongxin Li, Qiang Zhou, Qiang Wang, Jie Xiong

**Affiliations:** 1 Department of Urology, Aerospace Center Hospital, Beijing, China; 2 Department of Oncology, Suining Central Hospital, Suining, Sichuan, China; 3 Department of Systems Biology, City of Hope Comprehensive Cancer Center, Monrovia, CA, United States; 4 Department of Oncology, Qujing Central Hospital of Yunnan Province, Qujing, Yunan, China; 5 Department of Gastrointestinal Surgical Unit, Suining Central Hospital, Suining, Sichuan, China

**Keywords:** cancer progression, circRNA-protein interaction, circular RNA, metastasis, RNA-binding protein, therapy resistance, tumor metabolism, tumor microenvironment

## Abstract

Circular RNAs (circRNAs) are a class of endogenous non-coding RNAs characterized by covalently closed loop structures, which confer high stability and evolutionary conservation. Beyond their well-known role as microRNA sponges, circRNAs can directly interact with proteins to modulate protein stability, activity, subcellular localization, and transcriptional or epigenetic regulation. These circRNA–protein interactions play critical roles in cancer progression by influencing tumor cell proliferation, metastasis, stemness, metabolic reprogramming, cell death, and therapy resistance. Moreover, they can shape the tumor immune microenvironment, affecting immune cell infiltration, immune evasion, and responses to immunotherapy. Understanding the mechanisms and functional consequences of circRNA–protein interactions provides new insights into tumor biology and offers promising avenues for cancer diagnosis, prognosis, and therapeutic intervention.

## 1 Introduction

Circular RNAs (circRNAs) are a class of non-coding RNAs generated through back-splicing of precursor mRNAs, forming covalently closed loop structures. Lacking 5’ caps and 3’ polyadenylated tails, circRNAs exhibit remarkable stability, resistance to exonuclease-mediated degradation, and prolonged half-lives (1). These properties enable circRNAs to play critical roles in diverse physiological and pathological processes (1). In recent years, advances in high-throughput sequencing, circRNA-specific microarrays, and transcriptomics have revealed aberrant circRNA expression across multiple cancer types, implicating them in tumor proliferation, apoptosis, migration, invasion, stemness, and therapeutic resistance (1). Early studies primarily focused on the canonical function of circRNAs as microRNA (miRNA) sponges or competing endogenous RNAs (ceRNAs), regulating the expression of miRNA target genes to modulate tumor biology (2). However, accumulating evidence indicates that circRNAs can also directly interact with proteins, a mechanism referred to as circRNA–protein interaction (3). Such interactions influence protein stability, activity, subcellular localization, and transcriptional or epigenetic regulation (3). This mechanism not only broadens the functional repertoire of circRNAs but also positions them as central nodes within tumor signaling networks.

CircRNA–protein interactions exert multi-layered effects in cancer. For instance, they can regulate cell cycle proteins, apoptosis-related factors, or metabolic enzymes to modulate tumor cell proliferation and metabolic reprogramming (4). They can also interact with epithelial–mesenchymal transition (EMT)- or stemness-associated proteins to promote metastasis and maintain tumor-initiating cell properties. Moreover, circRNA–protein interactions can affect DNA damage repair pathways or drug target proteins, contributing to chemoresistance and radioresistance (4). Importantly, these interactions also modulate the tumor immune microenvironment, including immune cell infiltration, immune evasion, and responses to immunotherapy, offering potential novel targets for cancer immunotherapy (5).

Collectively, circRNA–protein interactions represent a crucial mechanism in tumorigenesis, progression, and immune regulation. They deepen our understanding of the molecular underpinnings of cancer and provide new avenues for the development of diagnostic biomarkers and therapeutic interventions (5). As research advances, systematic elucidation of circRNA–protein interaction networks is poised to become a frontier in cancer biology and precision medicine.

## 2 circRNA–protein interactions

CircRNA–protein interactions can regulate protein stability, localization, activity, and epigenetic functions, while proteins can also influence circRNA biogenesis.

### 2.1 circRNA regulates protein stability

**Figure 1:**
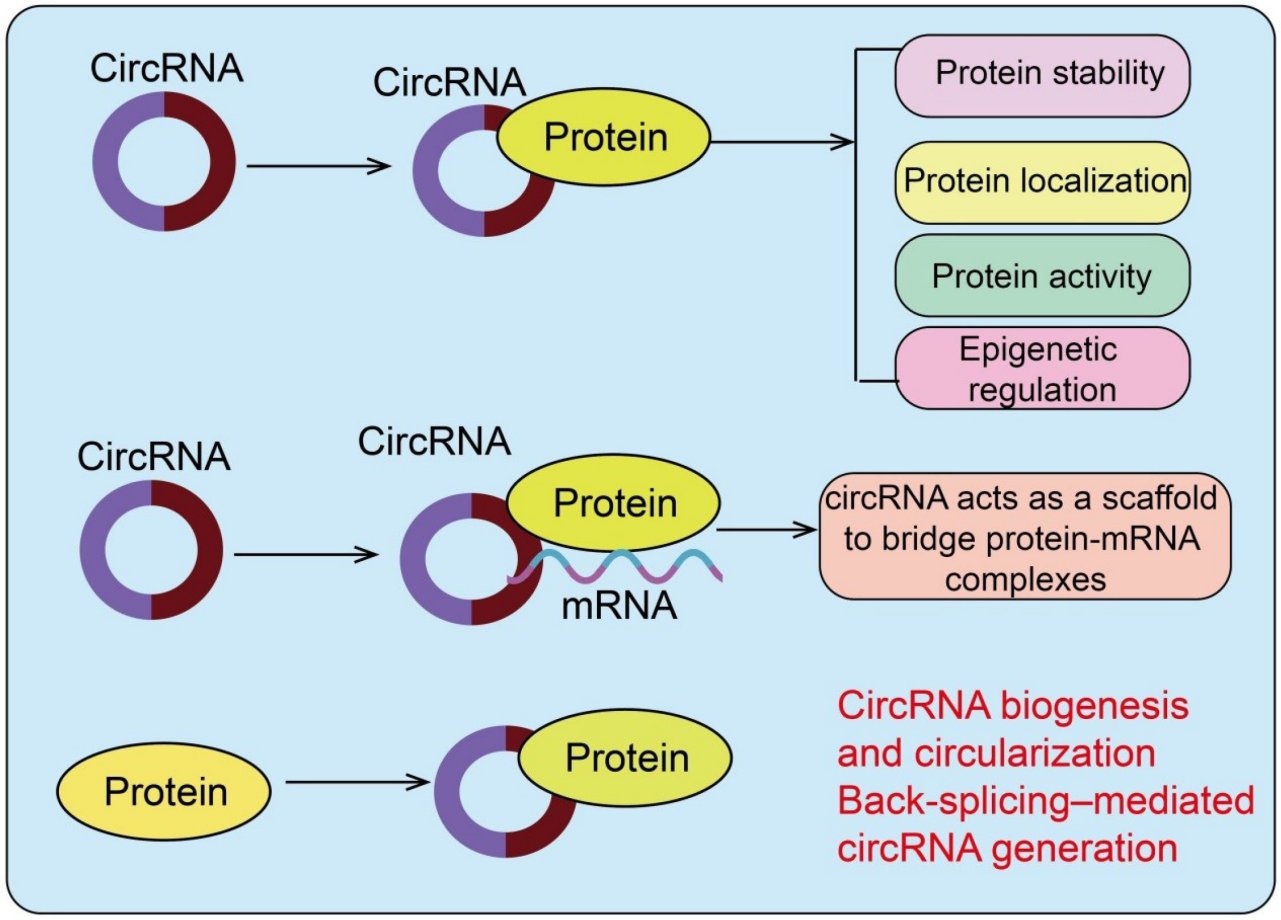
Schematic representation of circRNA–protein interactions. circRNAs can bind proteins to regulate protein stability, localization, activity, RNA interactions, and epigenetic regulation. Proteins can also influence circRNA biogenesis and circularization via back-splicing-mediated circRNA generation. CircRNAs also function as molecular scaffolds that bridge RNA-binding proteins with specific mRNA targets, thereby stabilizing transcripts.

CircRNAs can directly bind to proteins, thereby modulating their degradation or stability and influencing key intracellular signaling pathways. For example, circRNA-CREIT functions as a scaffold to facilitate E3 ubiquitin ligase HACE1-mediated degradation of PKR, regulating protein stability and enhancing the sensitivity of triple-negative breast cancer (TNBC) cells to doxorubicin (6) (**Figure 1**). This circRNA–protein interaction suppresses stress granule formation and activates apoptotic signaling, highlighting the critical role of circRNA-mediated protein stability in chemotherapy response. Similarly, circLRFN5 is downregulated in glioblastoma and correlates with poor patient prognosis. CircLRFN5 binds to PRRX2, promoting its ubiquitin-dependent proteasomal degradation, which reduces GCH1 transcriptional activity, induces ferroptosis in glioma stem cells, and suppresses proliferation, stemness maintenance, and tumorigenesis (7). These findings illustrate how circRNAs influence tumor cell fate by modulating protein stability. By stabilizing key proteins, circRNAs regulate tumor cell proliferation, apoptosis, and other biological processes, positioning them as potential therapeutic targets in cancer.

### 2.2 circRNA modulates protein activity

CircRNAs can directly interact with enzymes or signaling molecules, modulating their activity or complex formation and consequently altering downstream signaling pathways. In colorectal cancer (CRC), certain circRNAs are stabilized via METTL1-mediated m7G modification, predominantly at GG motifs. METTL1-mediated modification prevents circKDM1A degradation, thereby promoting CRC cell proliferation, invasion, and migration *in vitro* and *in vivo*, and activating the AKT pathway through PDK1 upregulation, driving tumor progression (8) (**Figure 1**). This indicates that m7G modification of circRNAs can influence protein signaling pathways and cancer development by regulating circRNA stability. FEACR directly binds to NAMPT, enhancing its stability and promoting NAMPT-dependent Sirt1 expression (9). This reduces FOXO1 acetylation, increases its transcriptional activity, and upregulates ferritin heavy chain 1 (Fth1), thereby inhibiting ferroptosis in cardiomyocytes (9). This circRNA–protein interaction plays a protective role in ischemia-reperfusion injury, improving cardiac function and revealing a novel mechanism by which circRNAs regulate cell fate via protein activity modulation.

### 2.3 circRNA regulates protein localization

Within cells, circRNAs can act as molecular guides to control the subcellular localization of proteins, influencing their distribution between the nucleus, cytoplasm, or organelles. Some circRNAs facilitate the nuclear translocation of key transcription factors or signaling molecules, thereby affecting gene expression. CircIPO7 is highly expressed in nasopharyngeal carcinoma (NPC) patients with distant metastases. Knockdown of circIPO7 inhibits NPC cell metastasis and enhances cisplatin sensitivity (10). Mechanistically, circIPO7 binds cytoplasmic YBX1, promoting AKT-mediated phosphorylation at Ser102, which drives YBX1 nuclear translocation and activates transcription of FGFR1, TNC, and NTRK1. Clinically, high circIPO7 expression correlates with poor distant metastasis-free survival in cisplatin-treated NPC patients, highlighting its role in tumor metastasis and chemotherapy response (10) (**Figure 1**). CircIMMP2L is upregulated in advanced esophageal squamous cell carcinoma (ESCC) and in patients with lymph node metastasis (LNM), correlating with poor prognosis and predictive of LNM even at T1 stage (11). Functional assays demonstrate that circIMMP2L knockdown inhibits malignant progression of ESCC. Cytoplasmic circIMMP2L binds CtBP1, promoting its nuclear retention independently of CtBP2, and enhances CtBP1–HDAC1 interactions, epigenetically repressing E-cadherin and p21 transcription (11). CircXRN2 is downregulated in bladder cancer, and its overexpression inhibits tumor cell proliferation and migration *in vitro* and *in vivo* while suppressing glycolysis and lactate production. Mechanistically, circXRN2 binds the SPOP degron to prevent LATS1 degradation, activating the Hippo pathway (12). Additionally, the circXRN2–Hippo axis modulates H3K18 lactylation and LCN2 expression, influencing bladder cancer progression. These findings illustrate that circRNAs fine-tune tumor cell behavior by controlling protein subcellular localization (12). Beyond acting as miRNA sponges, many circRNAs also function as molecular scaffolds that bridge RNA-binding proteins with specific mRNA targets, thereby stabilizing transcripts or modulating their translation (13). In NSCLC, circ0515 exemplifies this scaffold function by recruiting the RNA-binding protein RBM45 to form a protein–mRNA complex with SDHB mRNA, enhancing its stability and promoting mitochondrial oxidative phosphorylation and chemoresistance (**Figure 1**) (13).

### 2.4 circRNA influences transcription and epigenetic regulation

Nuclear circRNAs can interact with chromatin regulators or transcription factors, participating in transcriptional or epigenetic regulation. CircRNAs can modulate histone modifications or RNA polymerase complex activity, thereby regulating target gene expression. CircCFL1 is highly expressed in TNBC cells and tissues and exhibits prognostic potential (14). Functionally, circCFL1 promotes TNBC cell proliferation, metastasis, and stemness maintenance. Mechanistically, it serves as a scaffold to enhance HDAC1–c-Myc interaction, inhibiting c-Myc K48 ubiquitination and stabilizing its levels. Stabilized c-Myc binds the TP53 promoter to promote mutp53 expression, enhancing stemness via the p-AKT/WIP/YAP/TAZ pathway (14) (**Figure 1**). CircCFL1 also upregulates PD-L1, suppressing CD8^+^ T cell-mediated antitumor immunity. These findings reveal that circCFL1 exerts oncogenic effects via the HDAC1/c-Myc/mutp53 axis and may serve as a diagnostic marker and therapeutic target in TP53-mutant TNBC (14). CircLPAR1 is downregulated in CRC tissues and correlates with overall survival. Exosomal circLPAR1 internalized by CRC cells binds eIF3h, disrupting METTL3–eIF3h interaction, thereby reducing BRD4 translation and inhibiting tumor growth (15). This illustrates a novel mechanism by which circRNAs regulate cancer progression through protein interactions affecting transcription and translation.

### 2.5 m6A modification regulates circRNA expression

Across multiple cancer types, emerging evidence suggests that m6A modification may represent a recurrent but context-dependent regulatory layer in circRNA–protein interactions (16). Rather than acting as a universal determinant of circRNA–protein binding, m6A appears to modulate interaction specificity by influencing circRNA stability, subcellular localization, or protein affinity in a cancer- and pathway-dependent manner (16). This pattern underscores that overlapping regulatory modes, such as m6A modification, function as adaptable molecular enablers rather than pan-cancer drivers, emphasizing the necessity of interpreting circRNA–protein interactions within their specific cellular and immunological contexts. For example, m6A-modified circPAK2 undergoes YTHDC1-mediated cytoplasmic translocation and forms a circPAK2/IGF2BP/VEGFA complex to drive angiogenesis and metastasis (17), whereas circAURKA promotes CRC proliferation through stabilizing CTNNB1 via enhanced ACLY–CTNNB1 interaction (18). Similarly, circIGF2BP3 modulates the PKP3/OTUB1 axis to increase PD-L1 expression and suppress CD8^+^ T cell immunity in NSCLC (19). These studies collectively suggest that m6A functions as a context-dependent facilitator of circRNA–protein binding and subcellular localization, enabling diverse downstream effects across cancer types. To avoid redundancy and enhance thematic synthesis, we therefore treat m6A modification as a unifying regulatory module that shapes circRNA–protein interaction landscapes, while the specific oncogenic or immune-evasive outcomes remain cancer- and pathway-specific.

### 2.6 RNA-binding proteins regulate circRNA biogenesis

CircRNA biogenesis is regulated by RNA-binding proteins (RBPs), which can promote or inhibit pre-mRNA back-splicing, thereby determining circRNA abundance and species. CircNFIB is markedly upregulated in pancreatic ductal adenocarcinoma (PDAC) and perineural invasion (PNI) samples, and its overexpression promotes PNI *in vitro* and *in vivo*. Mechanistically, circNFIB binds IGF2BP3, enhancing L1CAM mRNA stability and activating ERK/MAPK signaling to drive PNI (20). RBMS1 binds NFIB pre-mRNA, facilitating circNFIB formation. CircNFIB also represents a potential therapeutic target, as its modulation reduces the antitumor efficacy of SCH772984 (20)(**Figure 1**). CircRPPH1 is upregulated in TNBC and associated with poor prognosis. Functionally, circRPPH1 promotes malignant phenotypes *in vitro* and *in vivo* by sponging miR-326 to regulate ITGA5 expression, activating the FAK/PI3K/AKT pathway. ZNF460 enhances circRPPH1 expression, forming a circRPPH1/miR-326/ITGA5 axis that drives TNBC progression (21). RBP–circRNA interactions thus form feedback regulatory networks with critical roles in tumor signaling and cell fate determination.

## 3 Functions and mechanisms of circRNA–protein interactions in cancer progression

**Figure 2:**
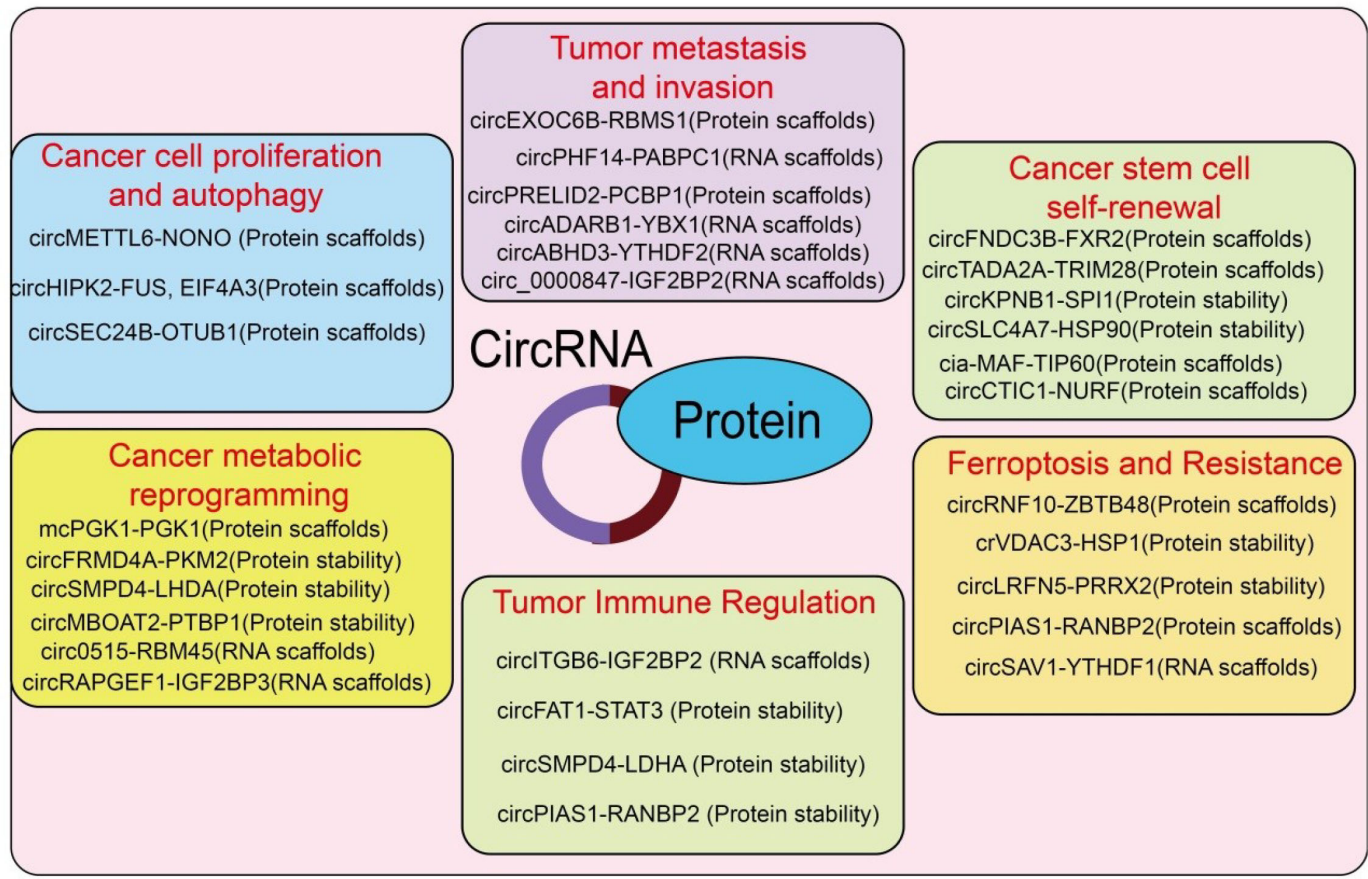
Overview of circRNA–protein interactions in cancer-related processes. This schematic summarizes representative circular RNAs (circRNAs) that modulate cancer progression through direct interactions with proteins, functioning as protein scaffolds, regulators of protein stability, RNA scaffolds. CircRNA–protein complexes are involved in diverse oncogenic processes, including cancer cell proliferation and autophagy, tumor metastasis and invasion, cancer stem cell self-renewal, cancer metabolic reprogramming, ferroptosis and therapy resistance, and tumor immune regulation. By organizing or stabilizing specific protein partners, circRNAs orchestrate signaling pathways and cellular programs that collectively drive tumor initiation, progression, immune evasion, and treatment resistance.

CircRNA–protein interactions orchestrate multiple aspects of tumor progression by modulating protein stability, activity, localization, and transcriptional or epigenetic regulation, thereby influencing proliferation, metastasis, stemness, therapy resistance, and the tumor microenvironment (**Figure 2**).

### 3.1 circRNA–protein interactions regulate tumor cell proliferation and autophagy

CircRNAs can bind proliferation-related proteins to regulate cell cycle, apoptosis, or signaling pathway activity, thereby promoting or inhibiting tumor cell growth. CPSF6 is upregulated in esophageal squamous cell carcinoma (ESCC) tissues, and its cytoplasmic overexpression correlates with poor prognosis. CPSF6 promotes tumor growth by enhancing cell proliferation and colony formation while regulating the cell cycle and apoptosis (22). Moreover, CPSF6 functions as a circRNA-binding protein interacting with circCPSF6 (hsa_circ_0000417) to regulate its function, and loss of circCPSF6 induces cell cycle arrest and apoptosis (22). CircHIPK2 is upregulated in both inflammatory bowel disease (IBD) and colorectal cancer (CRC); its silencing inhibits CRC cell growth and exacerbates inflammation while attenuating inflammation-related tumorigenesis in DSS-induced colitis. Mechanistically, FUS mediates the interaction between circHIPK2 and EIF4A3, promoting TAZ translation and upregulating downstream targets CCN1 and CCN2 via the Hippo pathway, highlighting circHIPK2’s role in shared pathogenic mechanisms of IBD and CRC (23) (**Figure 2**). CircMETTL6 is downregulated in ovarian cancer and correlates with poor prognosis (24). Overexpression of circMETTL6 inhibits cancer cell proliferation, migration, invasion, tumor growth, and metastasis *in vitro* and *in vivo* by binding NONO’s coiled-coil domain, blocking its interaction with POLR2A, and suppressing GDF15 transcription, revealing the circMETTL6/NONO/GDF15 axis as a potential therapeutic target (24) (**Figure 2**). CircATG7 enhances ATG7-dependent autophagy by cytoplasmic sponging of miR-766-5p and nuclear promotion of HUR/ATG7 mRNA binding, accelerating pancreatic cancer proliferation, migration, and tumor progression (25). CircSEC24B scaffolds OTUB1 and SRPX2 to stabilize SRPX2, activating autophagy and promoting CRC proliferation and chemoresistance (26) (**Figure 2**). CircRAPGEF5 binds IGF2BP2 KH3–4 domains to stabilize NUP160 mRNA, suppressing autophagy and promoting lung adenocarcinoma proliferation, migration, invasion, and tumor growth (27). Notably, circMETTL6–NONO, circHIPK2–FUS, and circRAPGEF5–IGF2BP2 do not converge on common canonical signaling hubs but instead exhibit pronounced tumor-type specificity, with their regulatory effects observed exclusively in the cancer contexts in which they were originally reported. These circRNA–protein interactions appear to function in a highly context-dependent manner, showing strong tumor-type specificity rather than acting as universal regulators across multiple cancer types.

### 3.2 circRNA–protein interactions regulate cancer metastasis

CircRNAs can interact with EMT or migration-related proteins, modulating cytoskeletal remodeling, adhesion, and migratory capacity to influence tumor metastasis. CircHERC1 is significantly upregulated in lung cancer tissues and cells, promoting proliferation, invasion, and metastasis while inhibiting apoptosis; its knockdown yields opposite effects (28). Cytoplasmic circHERC1 acts via two mechanisms: as a competitive endogenous RNA (ceRNA) sponging miR-142-3p to derepress HMGB1, activating MAPK/ERK and NF-κB pathways, and binding FOXO1 to retain it in the cytoplasm, modulating the AKT feedback pathway (28). CircEXOC6B, derived from the EXOC6B gene, inhibits prostate cancer (PCa) metastasis by scaffolding RBMS1 and HuR to upregulate AKAP12 (29) (**Figure 2**). Similarly, circHPS5, circCDYL, circPRELID2, circADARB1, circABHD3, and circ_0000847 regulate EMT, stemness, and metastasis through diverse protein interactions, m6A modifications, and subcellular localization, demonstrating the central role of circRNA–protein interactions in cancer invasion and dissemination (30).

### 3.3 circRNA–protein interactions maintain cancer stem cell self-renewal

CircRNAs can interact with stemness-regulating proteins, including Wnt/β-catenin or Notch pathway components, to maintain cancer stem cell (CSC) self-renewal. m6A-modified circFNDC3B, via YTHDC1-mediated cytoplasmic export, binds FXR2 to stabilize RNF41 expression, promoting ASB6 ubiquitination and suppressing CRC stemness and metastasis (31). CircTADA2A inhibits FLT3-ITD AML proliferation and stemness by disrupting TRIM28/MDM2 complex formation, activating p53 (32) (**Figure 2**). CircKPNB1 enhances SPI1 stability and nuclear translocation to activate TNF-α/NF-κB signaling in GBM, forming a DGCR8-dependent feedback loop that promotes glioblastoma stem cell (GSC) proliferation and stemness (**Figure 2**). Other circRNAs, including circRPPH1, circ-MALAT1, circIPO11, circSLC4A7, circCTIC1, hsa-circ_0003420, cia-MAF, and rtciscE2F (33), regulate CSC self-renewal through transcriptional, post-transcriptional, or epigenetic mechanisms, underscoring circRNA–protein interactions as critical modulators of tumor recurrence and therapy resistance.

### 3.4 circRNA–protein interactions reprogram tumor metabolism

CircRNAs can bind metabolic enzymes or transcription factors to regulate glycolysis, lipid metabolism, and amino acid metabolism. Mitochondria-derived circRNA mcPGK1 in liver tumor-initiating cells (TICs) promotes PGK1-mediated metabolic reprogramming, suppressing oxidative phosphorylation and enhancing glycolysis, increasing α-ketoglutarate and lactate, and activating Wnt/β-catenin signaling to sustain TIC self-renewal and tumorigenesis (34) (**Figure 2**). CircRREB1 and circFRMD4A modulate glycolysis and Wnt signaling in pancreatic cancer and breast cancer, respectively, affecting stemness and tumor growth (35, 36) (**Figure 2**). CircSIPA1L3, circSMPD4, circMBOAT2, circ0515, circABCA1, circRAPGEF1, and circSOBP similarly regulate metabolic enzymes or mRNA stability to reprogram glucose, lipid, and mitochondrial metabolism, supporting tumor growth, adaptation, and therapy resistance (37).

### 3.5 circRNA–protein interactions in ferroptosis and therapy resistance

CircRNA–protein interactions influence tumor cell survival and therapeutic response by modulating metabolic pathways, oxidative stress, DNA damage repair, and cell death programs. In glioma stem cells, circRNF10 promotes self-renewal and ferroptosis resistance through a circRNF10/ZBTB48/IGF2BP3 feedback loop (38), while exosomal circRNA_101093 (cir93) in LUAD stabilizes FABP3-mediated arachidonic acid metabolism, suppressing lipid peroxidation and ferroptosis (39) (**Figure 2**). Several other circRNAs, including circLRFN5 (7), circPIAS1 (40), circSAV1 (41), circSTIL (42), circFRMD4A (36), and circKIAA1797 (43), regulate ferroptosis or cuproptosis via protein interactions, post-translational modifications, or mRNA stabilization, thereby affecting tumor survival and therapy efficacy. Notably, many circRNA–protein interactions that confer therapy resistance converge on similar mechanisms as ferroptosis regulation, such as metabolic reprogramming and redox homeostasis. For instance, circRNA-SORE mediates sorafenib resistance in HCC by stabilizing YBX1 (44), and circRNA-CREIT enhances doxorubicin sensitivity in TNBC by scaffolding PKR and HACE1 to induce PKR degradation and apoptosis (6) (**Figure 2**). Together, these findings suggest that circRNA–protein interactions act as pivotal determinants of both ferroptosis and therapy resistance, offering potential targets for improving clinical outcomes.

## 4 circRNA–protein interactions regulate the tumor immune microenvironment

CircRNA–protein interactions orchestrate multiple aspects of the tumor immune microenvironment by modulating immune cell infiltration and function, facilitating cancer immune evasion, and influencing the efficacy of tumor immunotherapies.

### 4.1 circRNA–protein interactions regulate immune cell infiltration and function

circRNAs can bind to immune regulatory proteins, influencing the recruitment, activation, and functional state of immune cells, thereby modulating the tumor microenvironment. Exosomal circRNA_0013936 derived from bladder cancer inhibits CD8^+^ T cell function in PMN-MDSCs by sponging miR-320a and miR-301b, upregulating FATP2 and downregulating RIPK3 (45). In lung cancer, Kras-driven circRNAs (circHIPK3 and circPTK2) form an immunosuppressive network by upregulating M2 macrophages and monocyte-derived MDSC subsets (Gr1-/CD11b- and Gr1-/CD11b+), which not only promotes tumor immune evasion and chemoresistance but also enhances tumor heterogeneity and lymph node metastasis (46). Combined inhibition of circPTK2 and M2 macrophage signaling suppresses lung tumor growth and metastasis and prolongs survival. In breast cancer, circ-E-cadherin and its encoded protein C-E-cad are significantly upregulated; C-E-cad promotes CXCL8 transcription by activating EGFR signaling in tumor cells and directly supports PMN-MDSCs glycolysis, facilitating MDSC recruitment and survival (47). Targeting C-E-cad enhances anti–PD-1 immunotherapy efficacy. In ovarian cancer, circITGB6 is significantly elevated in tumor tissues and serum, correlating with cisplatin resistance and poor prognosis; circITGB6 forms a ternary complex with IGF2BP2 and FGF9 mRNA in the cytoplasm, stabilizing FGF9 mRNA and inducing M2 macrophage polarization, thereby promoting M2-dependent cisplatin resistance (48) (**Figure 2**). Antisense oligonucleotides targeting circITGB6 block M2 polarization and markedly reverse cisplatin resistance. In hepatocellular carcinoma (HCC), tumor-associated macrophages (TAMs) upregulate circMRCKα to promote tumor glycolysis and progression; circMRCKα encodes a 227-amino acid functional peptide (circMRCKα-227aa) that binds USP22, preventing ubiquitin-dependent degradation of HIF-1α, enhancing HCC glycolysis and malignancy (49). circMRCKα levels correlate positively with CD68^+^ TAM infiltration and USP22 expression and serve as an independent prognostic factor in HCC (49). circARAP2 (hsa_circ_0069396) is upregulated during NK cell desensitization and binds transcription factor CTCF, preventing the recruitment of the CTCF-PRC2 complex to the RAB5A promoter, removing H3K27 and H3K9 methylation-mediated repression, enhancing RAB5A transcription, and promoting sMICA-induced NKG2D endocytosis and NK cell desensitization (50). Inhibition of circARAP2 effectively alleviates NK cell desensitization, providing a potential therapeutic target for tumor immune evasion (50). Highly expressed circ_0001947 is significantly elevated in gastric cancer (GC) and its derived small extracellular vesicles (sEVs), correlating with poor prognosis; it sponges miR-661 and miR-671-5p to upregulate CD39, promoting CD8^+^ T cell exhaustion and immune tolerance (51). Inhibition of circ_0001947 alleviates CD8^+^ T cell exhaustion and enhances anti–PD-1 therapy response (51). These two examples highlight distinct, cell-type-specific modes by which circRNA-centered regulatory networks shape effector immune cell dysfunction within the tumor microenvironment. In NK cells, circARAP2 directly engages a transcription factor–chromatin regulatory axis by binding CTCF, thereby modulating PRC2-mediated histone methylation at the RAB5A promoter. This epigenetic reprogramming enhances RAB5A-dependent NKG2D endocytosis, leading to NK cell desensitization. In contrast, circ_0001947 primarily operates in CD8^+^ T cells through a post-transcriptional regulatory mechanism, indirectly sustaining immune checkpoint signaling by upregulating CD39 and promoting T cell exhaustion, which in turn affects responsiveness to PD-1 blockade. Together, these findings underscore that circRNA-associated immune regulation is highly context-dependent, with distinct molecular layers—epigenetic control in NK cells versus immune checkpoint–related metabolic and signaling regulation in T cells—governing effector cell dysfunction. High expression of circCCAR1 is observed in HCC tumor tissues, plasma exosomes, HCC cells, and culture supernatants; circCCAR1 sponges miR-127-5p to upregulate WTAP, forming a circCCAR1/miR-127-5p/WTAP positive feedback loop, accelerating HCC proliferation and metastasis (52). EP300 and EIF4A3 promote circCCAR1 biogenesis, and WTAP-mediated m6A modification enhances its stability through IGF2BP3 binding. Tumor-secreted circCCAR1 can be taken up by CD8^+^ T cells, stabilizing PD-1 protein and impairing its function, promoting anti–PD-1 resistance (52). Additionally, EP300-upregulated CCAR1 enhances CCAR1/β-catenin interaction, further promoting PD-L1 transcription. circRNA–protein interactions regulate immune cell behavior and significantly influence tumor immune surveillance.

### 4.2 circRNA–protein interactions regulate cancer immune evasion

circRNAs can bind inhibitory immune signaling molecules or transcription factors, blocking antitumor immune responses and facilitating immune evasion. In squamous cell carcinoma (SCC), circFAT1 is upregulated and promotes cancer stemness and immune evasion by activating STAT3. circFAT1 binds STAT3 in the cytoplasm, preventing SHP1-mediated dephosphorylation, maintaining STAT3 activation, and suppressing STAT1-mediated transcription (53) (**Figure 2**). Knockdown of circFAT1 reduces tumor stemness, activates innate immunity, and significantly enhances PD-1 blockade efficacy by promoting CD8^+^ T cell infiltration and improving the tumor microenvironment (53). In HCC, circSMPD4 is a novel circRNA that promotes tumor immune evasion and metastasis by inducing lactate metabolic reprogramming. In NK cell-driven tumor evolution models *in vitro*, circSMPD4 directly binds lactate dehydrogenase A (LDHA) and reduces its acetylation via SIRT2-dependent deacetylation, preventing degradation through chaperone-mediated autophagy-lysosome pathways, thereby maintaining lactate metabolism (54) (**Figure 2**). This study reveals the oncogenic role of circSMPD4 in tumor metabolic reprogramming and its new mechanism in promoting immune evasion and tumor progression, highlighting the key role of circRNA–protein interactions in tumor immune evasion.

### 4.3 circRNA–protein interactions regulate MHC-I antigen presentation and cytokine secretion

Emerging evidence indicates that circRNAs are not merely passive miRNA sponges or translational templates but can function as active post-transcriptional regulators by forming complexes with RNA-binding proteins, thereby fine-tuning immune-related gene expression and antigen presentation processes. In renal cell carcinoma (RCC), we demonstrate that circGRAMD4 directly interacts with the RBP RBM4, resulting in enhanced stability of the autophagic cargo receptor NBR1 mRNA (55). Elevated NBR1 expression subsequently promotes macroautophagy-dependent degradation of MHC-I molecules, leading to impaired antigen presentation on tumor cells (55). This mechanism represents a previously underappreciated upstream regulatory axis of the MHC-I pathway, whereby circRNA–protein complexes modulate antigen presentation not through canonical defects in antigen processing machinery, but via selective autophagic degradation of MHC-I, ultimately inducing CD8^+^ T cell dysfunction and facilitating tumor immune escape. In contrast, our circRNA-based cancer vaccine study in hepatocellular carcinoma (HCC) illustrates the immunostimulatory potential of engineered circRNAs. The circGPC3 vaccine enables sustained antigen expression due to the intrinsic stability of circular RNA, thereby continuously supplying antigenic substrates for immunoproteasome-mediated processing (56). When combined with a TLR4 agonist, innate immune signaling is further amplified, promoting cDC1 maturation and enhancing cross-presentation via the MHC-I pathway (56). This, in turn, strengthens cDC1–CD8^+^ T cell interactions and facilitates efficient initiation of adaptive immune responses, leading to robust reprogramming of the tumor microenvironment. Together, these findings from RCC and HCC models reveal a bidirectional regulatory role of circRNAs in antitumor immunity. Endogenous oncogenic circRNAs such as circGRAMD4 suppress MHC-I antigen presentation through circRNA–RBP–autophagy signaling, whereas therapeutic circRNAs such as circGPC3 enhance MHC-I–dependent immune activation by promoting antigen availability and dendritic cell function. This dual regulatory paradigm underscores circRNA–protein interactions as critical upstream modulators of antigen presentation and highlights circRNAs as both promising immunotherapeutic targets and vaccine platforms.

Although cytokine secretion is a key determinant of effective antitumor immunity, its upstream regulatory mechanisms are often insufficiently explored. circNOX4 is a CAF-specific circRNA that drives the conversion of normal fibroblasts into FAP^+^ CAFs in NSCLC through the miR-329-5p/FAP axis (57), thereby promoting tumor migration and invasion. This pathway further establishes an inflammatory fibroblast niche by inducing IL-6 secretion, and disrupting the circNOX4/IL-6 axis markedly suppresses tumor growth and metastasis (57). In this study, we extend the mechanistic understanding of immune suppression in HNSCC by demonstrating that the HPV-encoded circRNA circE7 functions as an epigenetic and metabolic regulator of T cell–associated cytokine signaling through a circRNA–protein interaction (58). Specifically, circE7 directly binds acetyl-CoA carboxylase 1 (ACC1), facilitating its dephosphorylation and enzymatic activation. Activated ACC1 alters intracellular acetyl-CoA flux, resulting in reduced H3K27 acetylation at the LGALS9 promoter and subsequent transcriptional repression of galectin-9. Galectin-9 is a critical upstream modulator of cytokine secretion through its interactions with the immune checkpoint receptors TIM-3 and PD-1 on CD8^+^ T cells (58). Suppression of LGALS9 expression driven by the circE7–ACC1 axis disrupts immune checkpoint–dependent signaling, leading to impaired secretion of cytotoxic cytokines such as IFN-γ and TNF-α, as well as increased T cell apoptosis. Thus, circE7 does not directly regulate cytokine genes but instead rewires cytokine secretion through metabolic-epigenetic control of immune checkpoint ligand expression. Collectively, these findings identify a previously unrecognized regulatory pathway in which a viral circRNA orchestrates immune evasion by coupling lipid metabolism, chromatin modification, and immune checkpoint signaling. This mechanistic framework highlights circRNA–protein complexes as upstream regulators of cytokine secretion and provides a strong rationale for combinatorial immunotherapy strategies targeting both PD-1 and TIM-3 in HPV-associated HNSCC.

### 4.4 circRNA–protein interactions regulate immune checkpoint molecule expression

Through direct interactions with RNA-binding proteins, circRNAs can regulate immune checkpoint molecule expression and inflammatory signaling cascades, ultimately shaping tumor immune evasion and the response to anti–PD-1/PD-L1 therapy. circPHLPP2 is upregulated in colorectal cancer (CRC) patients resistant to anti–PD-1 therapy; its overexpression promotes CRC cell proliferation and tumor growth, whereas knockdown enhances anti–PD-1 efficacy (59). Mechanistically, circPHLPP2 binds ILF3, promoting its nuclear accumulation, enhancing IL36γ transcription, reducing NK cell infiltration, and inhibiting granzyme B and IFN-γ production, thereby promoting tumor progression. This study uncovers a novel circRNA-mediated mechanism regulating CRC immune evasion, with circPHLPP2 serving as a prognostic marker and potential therapeutic target (59). circGRAMD4 is upregulated in RCC, binds RBM4 to stabilize NBR1 mRNA, promotes MHC-I degradation, suppresses CD8^+^ T cell function, and enables tumor immune evasion (55). circPETH is delivered via exosomes from tumor-associated macrophages to HCC cells; its encoded circPETH-147aa promotes glycolysis (60), ALDOA-S36 phosphorylation, and immunosuppression and can be inhibited by the small molecule Norathyriol to enhance CD8^+^ T cell function and anti–PD-1 efficacy. circCFL1 promotes TNBC cell proliferation, stemness maintenance, and PD-L1-mediated immune evasion via the HDAC1/c-Myc/mutp53 axis, impairing CD8^+^ T cell antitumor activity, suggesting its potential as a TNBC immunotherapy target (14). circPRDM4 recruits HIF-1α under hypoxia to promote PD-L1 transcription in HCC, enhancing CD8^+^ T cell immune evasion; its high expression correlates with anti–PD-1 therapy response, indicating potential as an immunotherapy regulatory factor.

Although most evidence for circRNA–protein interactions in immunotherapy currently comes from preclinical studies, a few early-stage clinical observations suggest translational potential. For example, circRNA signatures in circulating exosomes have been associated with response to immune checkpoint blockade in several cancer types, indicating that circRNAs may serve as predictive biomarkers for anti–PD-1/PD-L1 therapy. However, direct clinical trials targeting circRNAs are still limited, and more prospective studies are needed to validate their therapeutic value. Mechanistically, combining circRNA inhibitors with immune checkpoint blockade is supported by the dual roles of circRNA–protein interactions in both immune evasion and tumor-intrinsic survival pathways. Targeting circRNAs that stabilize PD-1 or suppress antigen presentation could directly enhance T cell recognition, while simultaneous PD-1/PD-L1 blockade could reinvigorate exhausted T cells. For example, circRHBDD1 stabilizes PD-L1 mRNA via IGF2BP2 in gastric cancer, inhibits CD8^+^ T cell infiltration, correlates with poor prognosis, and PLGA-PEG(si-circRHBDD1) combined with anti–PD-1 enhances immunotherapy efficacy (61). Therefore, rational combination strategies—such as circRNA-directed siRNA plus anti–PD-1 antibodies—may overcome resistance mechanisms and improve immunotherapy outcomes. Future clinical trials should prioritize circRNAs with well-defined protein partners and immune-relevant functions to maximize translational success.

### 4.5 CircRNA-encoded peptides are emerging as key mediators of cancer immunotherapy

In addition to acting as scaffolds or sponges, an increasing number of circRNAs have been shown to encode functional peptides, adding a new layer of regulatory complexity to circRNA-mediated immune modulation (62). These circRNA-derived peptides can directly engage canonical signaling pathways or epigenetic regulators, thereby reshaping the tumor immune microenvironment and influencing immunotherapy response. Given their unique biogenesis and often tumor-specific expression, circRNA-encoded peptides represent an emerging class of immunomodulatory molecules with potential as both biomarkers and therapeutic targets. Emerging evidence indicates that circRNA-encoded peptides represent a rapidly growing class of immunomodulatory effectors in cancer. For example, circPIAS1-108aa promotes STAT1 SUMOylation via recruitment of the SUMO E3 ligase Ranbp2, thereby inhibiting STAT1 phosphorylation, restoring SLC7A11/GPX4 signaling, and limiting IFNγ-induced immunogenic ferroptosis, which ultimately reduces the efficacy of immune checkpoint blockade (40). In contrast, circTNK2-487aa enhances STAT3 phosphorylation and suppresses STAT1 homodimer formation and CXCL10 expression, leading to impaired NK cell recruitment, while the circTNK2 RNA itself further activates AKT–mTOR signaling via SRSF1 binding (63). Overall, circRNA-encoded peptides expand the functional repertoire of circRNAs from RNA-level regulation to protein-level signaling control. By modulating key immune-related pathways such as STAT1/STAT3 signaling, ferroptosis, and chemokine expression, these peptides can directly determine immune cell recruitment, activation, and response to checkpoint blockade. Future studies should systematically profile circRNA-encoded peptides in immune-relevant contexts and clarify their specificity, stability, and immunogenicity, which will be crucial for translating this emerging class of molecules into immunotherapy strategies.

## 5 Therapeutic targeting of circRNA–protein interactions: opportunities and translational barriers

circRNA–protein complexes exhibit highly specific expression in cancers, demonstrating broad clinical potential (64). In diagnosis and prognosis, circRNA–protein complexes can be used for early cancer screening and therapeutic response prediction. Some complexes are detectable in blood, exosomes, or other body fluids, enabling non-invasive or minimally invasive detection, facilitating early diagnosis. Moreover, changes in their expression levels often correlate with tumor sensitivity to chemotherapy, targeted therapy, or immunotherapy. For example, the circRHBDD1–IGF2BP2 complex predicts anti–PD-1 treatment efficacy. circRNA–protein complex expression also correlates with tumor invasiveness, metastatic potential, and patient survival, serving as important references for patient risk stratification and individualized management (65). Therapeutically, circRNA–protein complexes provide new intervention targets. Small molecules, antibodies, or nucleic acid aptamers can be designed to block circRNA–protein interactions, inhibiting oncogenic signaling pathways. For tumor-suppressive circRNA–protein complexes, exogenous mimics may enhance their function, achieving antitumor effects. Additionally, delivery of circRNAs or interfering molecules to tumors via nanoparticles, liposomes, or exosomes improves targeting and efficacy while reducing systemic toxicity (66). Targeting circRNA–protein complexes to regulate immune checkpoint molecules like PD-L1 can enhance T cell or NK cell antitumor activity, providing new strategies for immunotherapy combinations.

Despite the promising therapeutic potential of targeting circRNA–protein interactions, several critical challenges must be addressed before clinical translation. First, off-target effects remain a major concern for circRNA-targeting strategies, particularly siRNAs and RNA interference approaches, which may cross-react with linear cognate transcripts or unintended RNA species due to sequence overlap (40). Additionally, delivery to the tumor microenvironment-especially to immune cells such as T cells, NK cells, and macrophages—poses a significant barrier, as current delivery platforms often lack sufficient specificity, stability, and tissue penetration. Second, the clinical utility of circRNA–protein complexes as biomarkers is limited by the lack of standardized detection methods. Although circRNAs can be enriched in extracellular vesicles and circulating blood, robust protocols for isolating and quantifying circRNA–protein complexes from clinical specimens remain underdeveloped, and reproducibility across platforms is still insufficient. Finally, the field currently suffers from publication bias and inconsistent findings, with few reports of negative or inconclusive results. A more balanced assessment of failed or contradictory studies is essential to avoid overestimation of therapeutic feasibility and to guide rational prioritization of targets. Addressing these challenges will require systematic evaluation of specificity, optimized delivery strategies, and standardized, clinically applicable detection assays to ensure reliable translation of circRNA–protein interaction biology into cancer immunotherapy.

## 6 Conclusion and perspectives

circRNA–protein interactions are critical regulators of tumorigenesis, metabolic reprogramming, and immune evasion, with significant clinical translational value. Future research can be advanced in three directions. First, systematic mapping of circRNA–protein interaction networks using high-throughput sequencing, mass spectrometry, and CLIP-seq can identify key oncogenic or tumor-suppressive complexes and cross-cancer comparisons may reveal broad-spectrum or tumor-specific targets (67). Second, further exploration of circRNA–protein complexes in the tumor immune microenvironment is warranted, including their roles in immune cell infiltration, immunosuppressive factor expression, and immune evasion mechanisms, to guide immune checkpoint inhibitor combination therapy and improve patient immune response rates. Finally, precise therapeutic strategies targeting circRNA–protein complexes can be developed by optimizing nucleic acid drugs, nanodelivery systems, and small molecule inhibitors to achieve efficient, low-toxicity treatment while exploring synergistic effects with chemotherapy, targeted therapy, and immunotherapy. In summary, circRNA–protein interactions not only provide new insights into tumor molecular mechanisms but also offer innovative approaches for early cancer diagnosis, therapeutic response prediction, and precision treatment, and are expected to become an important direction in clinical cancer translational research.
